# Engineering Antifouling Polysulfone Membranes Enhanced with Hydroxylated Amino-Functionalized TiO_2_ Nanotubes for Superior Water Filtration

**DOI:** 10.3390/polym18091096

**Published:** 2026-04-30

**Authors:** Ibrahim Hotan Alsohaimi, Mosaed S. Alhumaimess, Abdulelah Nashmi Alrashidi, Hassan Alwael, Meshal Alzaid, Mohamed R. El-Aassar, Ahmed A. Alshahrani, Hamud A. Altaleb, Hassan M. A. Hassan

**Affiliations:** 1 Chemistry Department, College of Science, Jouf University, Sakaka 2014, Saudi Arabia; mosaed@ju.edu.sa (M.S.A.); mrelaassar@ju.edu.sa (M.R.E.-A.); hmahmed@ju.edu.sa (H.M.A.H.); 2 Department of Chemistry, Faculty of Science, King Abdulaziz University, Jeddah 21589, Saudi Arabia; aalrashidi0080@stu.kau.edu.sa (A.N.A.); halwael@kau.edu.sa (H.A.); 3 Department of Physics, College of Science, Jouf University, Sakaka 2014, Saudi Arabia; mmalzaid@ju.edu.sa; 4 Water Desalination Technologies Institute, King Abdulaziz City for Science and Technology, Riyadh 11442, Saudi Arabia; ashahrni@kacst.edu.sa; 5 Department of Chemistry, Faculty of Science, Islamic University of Madinah, Madinah 41477, Saudi Arabia; haltaleb@iu.edu.sa

**Keywords:** titanium nanotubes, TNT@OH, ultrafiltration, antifouling, PSM membrane, water treatment, humic acid

## Abstract

Developing membranes with superior antifouling properties is crucial for efficient and sustainable water treatment. In this study, polysulfone (PSM) composite membranes were fabricated by incorporating hydroxylated titanium nanotubes (TNT@OH) via the non-solvent-induced phase separation method. The hydroxylation of TNTs enhanced their dispersion in the polymer matrix and promoted strong polymer–nanoparticle interactions. Comprehensive characterization using FTIR, XRD, TGA, FESEM, and AFM confirmed the successful integration of TNT@OH, resulting in membranes with improved hydrophilicity, porosity, and thermal stability. The contact angle decreased from ~88° for neat PSM to ~50° at 7 wt% TNT@OH, while surface free energy increased significantly. Mechanical strength and flexibility were also enhanced at optimal TNT@OH loadings (3–5 wt%), owing to uniform dispersion and strong interfacial bonding. Filtration experiments using humic acid (HA) and natural organic matter (NOM) demonstrated remarkable improvements in water flux, rejection efficiency, and fouling resistance. The composite membranes achieved HA rejection rates of up to 98%, with reduced irreversible fouling and higher flux recovery ratios across multiple filtration–cleaning cycles. The proposed antifouling mechanism is attributed to the formation of a stable hydration layer by surface hydroxyl groups, which prevents foulant adhesion and facilitates cleaning. These findings suggest that incorporating TNT@OH into polysulfone membranes is a promising approach for developing high-performance ultrafiltration membranes with enhanced permeability, mechanical robustness, and long-term antifouling stability, thereby making them suitable for advanced water purification applications.

## 1. Introduction

Membrane-based separation processes, particularly ultrafiltration (UF), have garnered significant attention for water and wastewater treatment due to their high efficiency in removing suspended solids, pathogens, dissolved organic matter, and other contaminants. These processes are energy-efficient compared to traditional therapies and produce high-quality water suitable for reuse and portable applications [1,2,3,4]. Among various polymeric materials used for membranes, polysulfone stands out due to its excellent thermal stability, mechanical robustness, and resistance to chemical degradation, making it widely used for UF membranes [5]. However, the hydrophobic nature of polysulfone membranes limits their performance by increasing their propensity to foul.

Membrane fouling is one of the most critical challenges in water treatment applications. Fouling occurs due to the accumulation of organic matter, microorganisms, colloidal particles, and inorganic scale on the membrane surface or within its pores. Organic fouling, particularly caused by humic substances and natural organic matter (NOM), is a major issue during surface water treatment. Fouling results in a significant reduction in permeate flux, increased transmembrane pressure, frequent cleaning, and ultimately, higher operational costs [6,7,8,9,10]. Traditional cleaning methods often fail to fully restore performance, leading to irreversible fouling and reduced membrane lifespan. As a result, strategies to enhance membrane hydrophilicity and reduce organic adsorption are being actively pursued [11]. Over the past decade, nanomaterials have emerged as effective additives to improve the surface properties of polymeric membranes. Materials such as titanium dioxide (TiO_2_) [12], graphene oxide (GO) [13,14], carbon nanotubes (CNTs) [15], and silica nanoparticles have been incorporated into PSF membranes to increase surface hydrophilicity, enhance water flux, and reduce fouling [16,17]. TiO_2_-based nanomaterials are especially attractive due to their photocatalytic activity, antibacterial properties, and strong hydrophilic nature [18]. Incorporating such nanoparticles can improve flux recovery ratios (FRR) and reduce irreversible fouling, thereby extending membrane life and lowering operational costs [19]. Titanium nanotubes (TNTs) are one-dimensional nanostructures characterized by high aspect ratios, large surface areas, and excellent mechanical properties. Their surfaces can be modified with hydroxyl groups (TNT@OH) to further increase hydrophilicity and facilitate strong interactions with the polymer matrix through hydrogen bonding [20,21,22,23]. TNT@OH provides improved dispersion in the casting solution, reducing nanoparticle agglomeration and resulting in uniform pore formation in the membrane. Studies have demonstrated that TNT incorporation reduces water contact angles, increases porosity, and enhances water permeability [24,25]. Additionally, TNT-based membranes have demonstrated superior antifouling behavior, with higher flux recovery and lower total fouling ratios than pristine polysulfone membranes [26]. While several studies have explored the use of TiO_2_ nanoparticles and TNTs for membrane modification, little research has examined hydroxylated TNTs (TNT@OH) combined with polysulfone for ultrafiltration membranes. The hydroxylation process is expected to improve nanoparticle dispersion and strengthen polymer–nanoparticle interactions by introducing additional surface–OH groups that promote hydrogen bonding with the polysulfone matrix. These hydroxyl groups enhance compatibility between the inorganic and organic phases, preventing nanotube agglomeration and resulting in a more uniform distribution in the casting solution. Improved dispersion creates membranes with more homogeneous pore structures and larger effective surface areas for water transport. As a result, hydroxylation can significantly enhance membrane hydrophilicity, increase water flux, and improve antifouling performance by reducing the adsorption of organic foulants and facilitating easier cleaning during filtration cycles.

This study aims to fabricate PSM-TNT@OH composite membranes with varying TNT@OH concentrations using the phase inversion method and evaluate their structural, mechanical, and filtration properties. The specific objectives include investigating changes in surface morphology, hydrophilicity, water permeability, humic acid (HA) and NOM rejection, and antifouling performance across repeated filtration cycles. This study presents a novel strategy for fabricating antifouling polysulfone membranes by incorporating hydroxylated titanium nanotubes (TNT@OH) as a highly hydrophilic nanofiller. Compared to conventional TiO_2_ nanoparticles, TNT@OH offers a higher density of surface hydroxyl groups and improved compatibility with the polymer matrix, leading to superior dispersion and stronger interfacial interactions. The key scientific contributions of this work include the systematic optimization of TNT@OH loading, the development of membranes with significantly enhanced hydrophilicity and porosity, and the demonstration of improved antifouling performance, including higher water flux, reduced organic fouling, increased flux recovery ratio (FRR), and enhanced removal of humic acid (HA) and natural organic matter (NOM). These findings provide new insights into nanofiller–polymer interactions and offer a promising pathway for designing next-generation high-performance membranes for sustainable water treatment applications.

## 2. Materials and Methods

### 2.1. Chemicals

3-aminopropyltriethoxysilane (APTEOS, ≥98%), NaOH (≥95%), polyvinyl pyrrolidone (PVP), HCl (36%), N, N-Dimethylformamide (≥99%), NaNO_2_ (≥97%), N-methyl-2-pyrrolidone (NMP, 99%), Polysulfone (PS, Mw ~35,000 by LS), CaCl_2_.2H_2_O (98%), NaCl, (99.5%), alginic acid sodium salt (SA, ≥99.5%), bovine serum albumin (BSA, ≥98%), and humic acid (HA, 226.14 g/mol) were delivered from Sigma-Aldrich Co., Ltd., St. Louis, MO, USA. The NOM solution was prepared following a previously reported method [27]. Briefly, bovine serum albumin (BSA), sodium alginate (SA), and humic acid (HA) were each added at a concentration of 10 mg L^−1^ to deionized (DI) water containing 1 mM CaCl_2_ and 7 mM NaCl. Deionized water was used throughout all preparation and measurement procedures.

### 2.2. Materials Preparation

#### 2.2.1. Synthesis of Aminated TiO_2_ Nanotubes (TNT@NH_2_)

A two-step process was used to prepare TNT@NH_2_. First, 1 g of TiO_2_ powder was dispersed in 50 mL of 10 M NaOH solution, stirred at room temperature, and then subjected to hydrothermal treatment at 140 °C for 72 h. The product was washed with 0.1 M HCl for 24 h, repeatedly rinsed with deionized water until the pH attained neutrality, and then dried at 110 °C for 24 h to obtain TiO_2_ nanotubes (TNTs). Second, 1.0 g of TiO_2_ tubes was mixed with 1 mL of APTEOS in 50 mL of toluene and then refluxed at 140 °C for 48 h under nitrogen. The mixture was subsequently washed with ethanol and dried at 80 °C for 24 h to yield aminated TNTs (TNT@NH_2_).

#### 2.2.2. Fabrication of Hydroxylated TiO_2_ NTs (TNT@OH)

To hydroxylate TNTs, 0.5 g of NaNO_2_ dissolved in 20 mL of water was added dropwise to a flask containing 1 g of TNT@NH_2_ in 20 mL of water, while stirring at 0 °C for 30 min to convert the amino groups into a diazonium derivative. The mixture was then boiled for 1 h to produce TNT@OH. The product was separated, washed with ethanol and deionized water, and vacuum-dried at 80 °C for 24 h.

#### 2.2.3. Fabrication of PSM-TNT@OH Composite Membranes

**Scheme 1:**
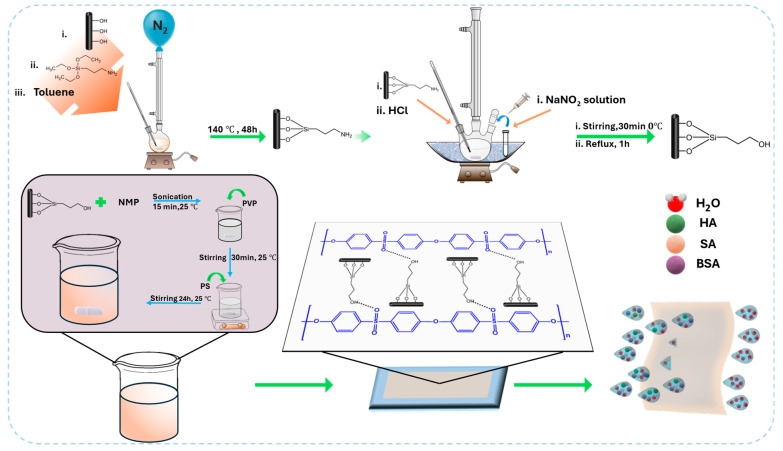
Visual representation of the fabrication process for PSM-TNT@OH membranes.

Composite membranes were fabricated via the non-solvent-induced phase separation (NIPS) method [28,29,30,31]. TNT@OH was first dispersed in NMP by sonication for 1 h, followed by the gradual addition of polysulfone (18 wt%) and PVP (2 wt%) under continuous stirring at 45 °C for 3 h. The resulting solution was further stirred for 24 h and degassed by sonication to remove trapped air bubbles. The homogeneous casting solution was then spread onto a glass plate using a casting knife with a fixed thickness of 250 µm. After a controlled evaporation (gelation) time of 20 s, the cast film was immediately immersed in a deionized water coagulation bath to induce phase separation (Scheme 1). After fabrication, the membranes were rinsed with deionized water and designated as PSM-TNT@OH (x), where ‘PSM’ refers to polysulfone membranes and ‘x’ represents the TNT@OH loading (1, 3, 5, or 7 wt%).

### 2.3. Characterizations

The synthesized materials were characterized using multiple analytical techniques. Fourier-transform infrared (FT-IR) spectra were recorded on a Shimadzu IR Tracer-100 spectrophotometer in the range of 4000–400 cm^−1^ to identify functional groups. Thermal stability was evaluated by thermogravimetric analysis (TGA) using a Shimadzu STA-449F3 instrument, with samples heated from 50 to 600 °C at 10 °C/min under an air atmosphere. Morphological features were examined by field emission scanning electron microscopy (FESEM) using a Thermo Scientific Quattro ESEM (Thermo Fisher Scientific Inc., Madrid, Spain). Surface hydrophilicity was determined through contact angle measurements with a Data Physics SCA20 goniometer (Filderstadt, Germany), while mechanical properties were assessed via tensile strength experiments conducted on a Shimadzu EZ-S tester. The surface free energy (SFE) of the prepared membranes was evaluated using the Owens–Wendt–Rabel–Kaelble (OWRK) approach, which separates the total surface energy (γ_s_) into its polar (γ_s_ᵖ) and dispersive (γ_s_ᵈ) contributions. The overall SFE was determined based on the measured contact angle (θ) values by applying the Owens–Wendt equation (Equation (1)).

*γ_L_*(1 + cos *θ*) = 2((*γ^d^_S_γ^d^_L_*)^1/2^ + (*γ^p^_S_γ^p^ _L_*)^1/2^) (1)

In this context, γₗ and γ_s_ correspond to the surface tensions of the liquid and solid, respectively. The overall surface free energy (γ_s_) was determined by summing its two components, namely the polar and dispersive contributions [32,33].

The membrane porosity (ε) was evaluated using the relationship:

(2) $$\varepsilon =\frac{W_{\mathrm{wet}}-W_{\mathrm{dry}}}{A\times t\times \rho }$$

where $W_{\mathrm{wet}}$ and $W_{\mathrm{dry}}$ correspond to the membrane weights in the saturated and dry conditions, respectively. The parameter A denotes the effective membrane area, t represents the membrane thickness, and ρ is the density of water. Subsequently, the water uptake (%) was determined according to:

(3) $$\phi =\frac{W_{\mathrm{wet}}-W_{\mathrm{dry}}}{W_{\mathrm{dry}}}$$

Here, $W_{\mathrm{wet}}$ and $W_{\mathrm{dry}}$ refer to the wet and dry weights of the membrane (kg), respectively. These parameters reflect the membrane’s ability to absorb and retain water, which is closely related to its hydrophilicity and internal pore architecture.

### 2.4. Evaluation of Ultrafiltration Performance

Membrane efficacy was tested in a dead-end UF cell operated at 1 bar, pH 7, and 25 °C. A 100 ppm humic acid (HA) solution in 800 mL deionized water served as the model foulant. The pure water flux (J_w_) was first measured using:

(4) $$J_{w}=\frac{m}{\rho \times A\times t}$$

where m is permeating mass, ρ is water density, A is the effective membrane area, and t is filtration time. During HA filtration, flux decline was monitored, and after cleaning, the recovered water flux (J_w2_) was recorded. Rejection (R_f_) was measured as:

(5) $$R_{f}\left(\%\right)=\frac{\left(C_{f}^{0}-C_{p}^{t}\right)}{C_{f}^{o}}\;\times 100$$

where C_f_ and C_p_ are the feed and permeate concentrations. The flux recovery ratio (FRR) was obtained as:

(6) $$FRR\left(\%\right)=\frac{Jw_{2}}{Jw_{1}}\times 100$$

Fouling behavior was further analyzed by determining reversible fouling (R_r_), irreversible fouling (R_ir_), and total fouling (R_t_):

(7) $$\;R_{r}=\frac{{(J}_{w2}-J_{fx})}{J_{w1}}\times 100$$

(8) $$R_{t}=\frac{{(J}_{w1}-J_{fx})}{J_{w1}}\times 100$$

(9) $$R_{ir}=\frac{{(J}_{w1}-J_{w2})}{J_{w1}}\times 100$$

Here, J_w1_ is the initial pure water flux, J_fx_ is the flux during HA filtration, and J_w2_ is the flux after cleaning.

The adsorption capacity of the membranes toward different foulants was investigated at pH 7. For this purpose, membrane samples (1 × 1 cm) were immersed in 10 mL of foulant solutions (100 mg L^−1^) contained in glass vials. The pH of each solution was adjusted to 7 using 0.1 M NaOH or HCl solutions. The vials were then placed on a shaker and maintained under continuous agitation at room temperature for 24 h to ensure equilibrium. After the adsorption period, the membranes were removed, and the residual HA foulant concentrations in the solutions were analyzed using an Agilent Cary 60 UV–Vis spectrophotometer. The amount of adsorbed foulant was determined based on the difference between the initial and final concentrations of the solutions. All experiments were conducted in triplicate, and the average values were reported. The adsorption capacity (q) of the membranes was calculated using the following equation:

(10) $$q=\frac{\left(C_{o}-C_{t}\right)\times V}{A}$$

where $C_{0}$ and $C_{t}$ represent the initial and final concentrations of the foulant solution, respectively, V is the solution volume, and A is the effective membrane area used in the adsorption test.

## 3. Results and Discussion

### 3.1. Characterization of TNT@OH

**Figure 1:**
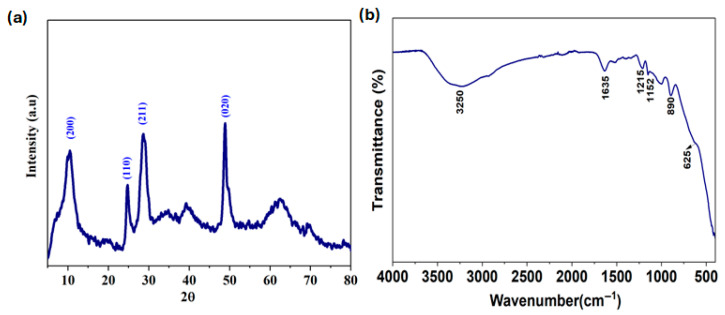
XRD patterns (**a**) and FTIR spectra (**b**) of synthesized TNT@OH powder.

Figure 1 presents the structural and chemical characterization of the synthesized TNT@OH powder using X-ray diffraction (XRD) and Fourier transform infrared spectroscopy (FTIR), confirming the successful formation and functionalization of titanium nanotubes. The XRD pattern shown in Figure 1a exhibits distinct diffraction peaks at approximately 2θ ≈ 10°, 24°, 28°, and 48°, which are indexed to the (200), (110), (211), and (020) planes, respectively. These characteristic peaks are consistent with the layered titanate nanotube structure, indicating the successful transformation of TiO_2_ into tubular titanate phases during the hydrothermal treatment. The relatively broad peaks suggest a nanoscale crystalline structure, which is typical for TiO_2_-derived nanotubes. The absence of additional impurity peaks confirms the high purity of the synthesized TNT@OH material. Moreover, the presence of the low-angle peak around 10° is particularly indicative of the interlayer spacing associated with nanotubular titanate structures, further validating the successful formation of nanotubes rather than bulk TiO_2_ particles. The FTIR spectrum in Figure 1b provides further insight into the surface functional groups of TNT@OH. The broad absorption band observed at around 3250 cm^−1^ corresponds to the stretching vibrations of hydroxyl (–OH) groups and adsorbed water molecules, confirming the hydroxylation of the nanotube surface. The peak at approximately 1635 cm^−1^ is attributed to the bending vibration of H–O–H groups, indicating the presence of physically adsorbed water. Additionally, the bands observed at 1215 and 1152 cm^−1^ are associated with Ti–O–Ti stretching vibrations, while the peak near 890 cm^−1^ corresponds to Ti–O–H bending, further confirming the presence of surface hydroxyl groups. The strong absorption band around 625 cm^−1^ is characteristic of Ti–O lattice vibrations, which is a signature of the titanium oxide framework. The combined XRD and FTIR results clearly demonstrate the successful synthesis of hydroxylated titanium nanotubes with a well-defined crystalline structure and abundant surface hydroxyl functionalities. These hydroxyl groups are expected to play a crucial role in enhancing the dispersion of TNT@OH within the polymer matrix, improving interfacial compatibility, and promoting membrane hydrophilicity. Consequently, this structural and chemical modification is anticipated to significantly enhance water permeability and antifouling performance in the fabricated composite membranes.

### 3.2. Membrane Investigation

**Figure 2:**
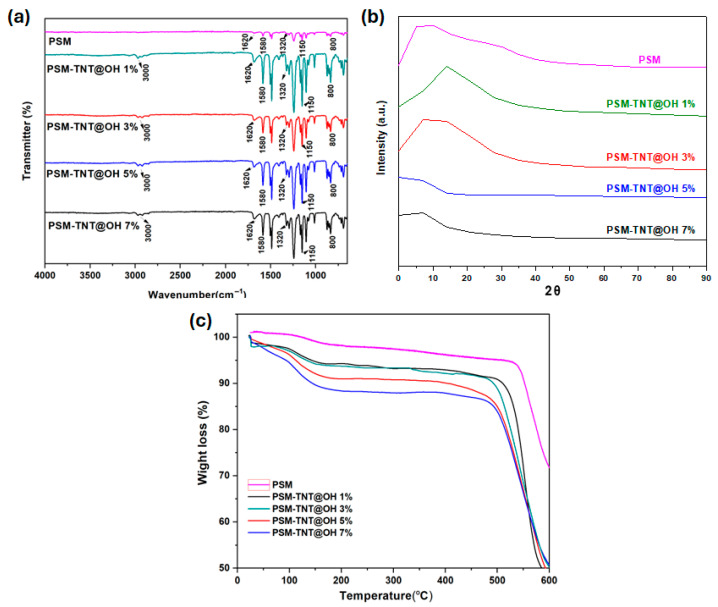
(**a**) FTIR spectra of neat PSM and composite membranes PSM-TNT@OH containing 1%, 3%, 5%, and 7% TNT@OH; (**b**) XRD patterns of neat PSM and the corresponding composite membranes; (**c**) TGA curves of the hybrid membranes with varying TNT@OH content (1–7 wt%) in the temperature range of 50–600 °C.

The FTIR spectra (Figure 2a) provide insight into the functional groups present in neat PSM and in hybrid membranes with varying TNT@OH loadings (1%, 3%, 5%, and 7%). Characteristic peaks of polysulfone, including aromatic C–H stretching (~3000 cm^−1^), C=C stretching of the phenyl ring (~1580 cm^−1^), and symmetric/asymmetric stretching of sulfone (–SO_2_–) groups (~1320 cm^−1^ and ~1150 cm^−1^), are observed in all samples, confirming that the polymer backbone remains structurally intact after TNT@OH incorporation. With increasing TNT@OH content, new absorption bands emerge or intensify near 3400 cm^−1^ (O–H stretching vibrations), 1620 cm^−1^ (Ti–O–H bending), and below 800 cm^−1^ (Ti–O stretching). These changes verify the successful introduction of hydroxylated titanium nanotubes into the polymer matrix. The stronger O–H peak intensity with higher TNT@OH loading indicates an increased number of surface hydroxyl groups, which enhance membrane hydrophilicity by improving hydrogen bonding with water molecules [20]. Such chemical modifications are consistent with previous studies showing that nanofiller incorporation improves wettability and antifouling properties [16].

The XRD analysis (Figure 2b) highlights the structural changes due to TNT@OH incorporation. The neat PSM exhibits a broad halo, typical of amorphous polymers, reflecting its non-crystalline nature. Upon TNT@OH addition, distinct diffraction peaks appear around 25° and 48°, corresponding to the (101) and (200) planes of anatase TiO_2_. The intensity of these bands rises with higher TNT@OH loadings, indicating greater amounts of crystalline TNT@OH embedded in the polymer matrix. This confirms that TNT@OH retains its anatase crystal structure during membrane fabrication and is well-dispersed in the polysulfone phase [24]. Such crystalline features are crucial because the anatase phase of TiO_2_ is known for its hydrophilicity and photocatalytic activity, both of which can improve antifouling performance [18].

The TGA profiles presented in Figure 2c illustrate the thermal stability of neat PSM and PSM–TNT@OH composite membranes with varying TNT@OH loadings. All membranes exhibit a similar degradation trend, characterized by an initial minor weight loss below ~150 °C, which can be attributed to the removal of physically adsorbed moisture and residual solvents. The main thermal degradation occurs in the temperature range of approximately 450–580 °C, corresponding to the decomposition of the polysulfone backbone. Compared to neat PSM, the incorporation of TNT@OH results in noticeable changes in the thermal behavior. The composite membranes show a slightly earlier onset of weight loss at intermediate temperatures, which may be associated with increased hydrophilicity and the presence of surface hydroxyl groups that facilitate moisture retention. However, at higher temperatures, the composite membranes exhibit improved thermal resistance, as evidenced by their higher residual weights at 600 °C. This enhanced char formation is attributed to the presence of thermally stable inorganic TNT@OH nanotubes, which act as heat barriers and restrict the mobility of polymer chains during thermal decomposition. Interestingly, the thermal stability does not increase linearly with TNT@OH content. While moderate loadings (1–3%) show comparable or slightly improved stability relative to neat PSM, higher loadings (5–7%) lead to a marginal reduction in thermal resistance at earlier stages of degradation, likely due to nanoparticle aggregation and the formation of localized defects within the polymer matrix [16,19]. Nevertheless, all composite membranes retain higher residual mass than neat PSM at elevated temperatures, confirming the reinforcing effect of TNT@OH. Overall, the TGA results, in conjunction with FTIR and XRD analyses, confirm the successful incorporation of TNT@OH into the PSM matrix. The presence of hydroxyl-functionalized nanotubes enhances interfacial interactions and improves thermal stability at high temperatures, while also influencing degradation behavior at intermediate temperatures. These structural and thermal modifications are expected to enhance membrane durability and performance under operating conditions.

**Figure 3:**
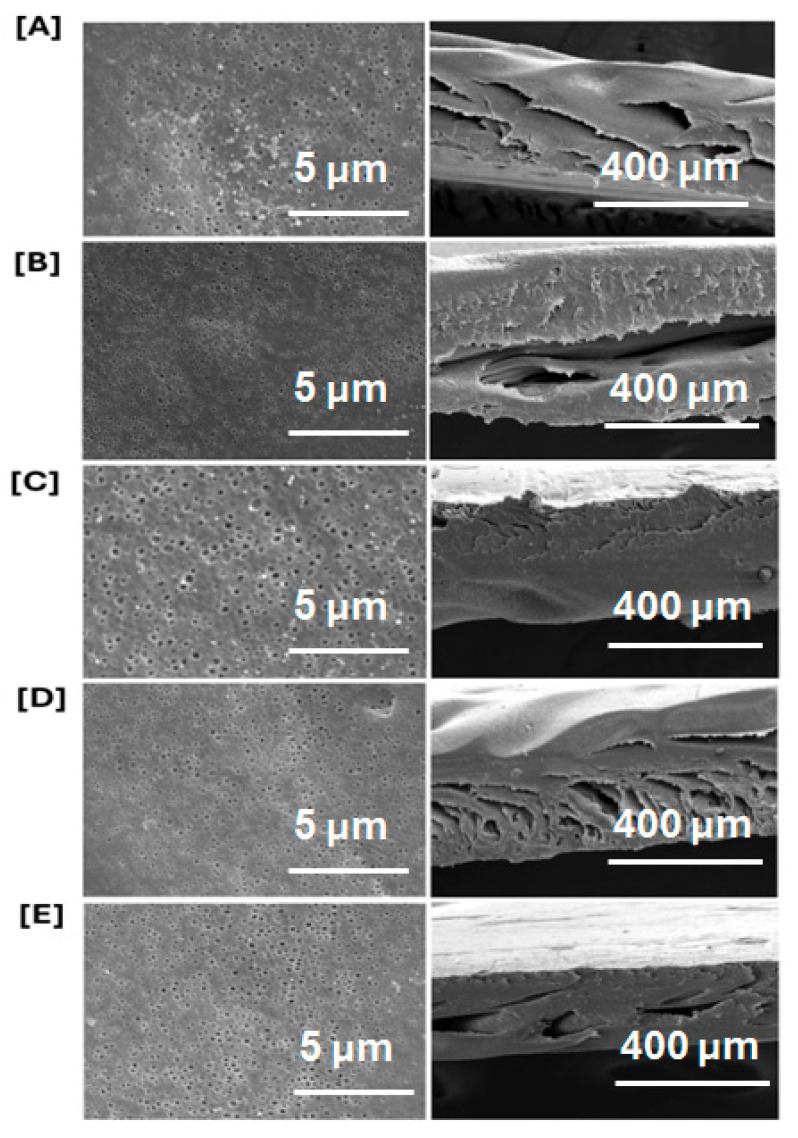
Top surface and cross-sectional FESEM images of hybrid membranes containing varying amounts of TNT@OH: (**A**) neat PSM; (**B**) PSM-TNT@OH 1%; (**C**) PSM-TNT@OH 3%; (**D**) PSM-TNT@OH 5%; and (**E**) PSM-TNT@OH 7%.

The FESEM images in Figure 3 were used to qualitatively examine the surface and cross-sectional morphology of the fabricated membranes. The top-surface image of the neat PSM membrane (Figure 3A) shows a relatively compact surface with small and sparsely distributed openings. After incorporating TNT@OH, noticeable changes in the surface morphology are observed. At 1 wt% loading (Figure 3B), the surface remains fairly uniform, although a slight increase in the number of surface pores can be distinguished. At 3 wt% TNT@OH (Figure 3C), the membrane exhibits a more open surface with clearer and more uniformly distributed pores. A similar porous surface is observed for the 5 wt% membrane (Figure 3D), whereas the 7 wt% membrane (Figure 3E) still exhibits surface pores but with a more compact, distinct appearance than the 3 wt% sample. These observations suggest that the incorporation of TNT@OH affects the phase inversion process and promotes the development of surface pores at moderate loadings, most likely due to the additive’s hydrophilicity, which enhances solvent–non-solvent exchange during membrane formation [22]. The cross-sectional FESEM images indicate that all membranes possess a dense upper skin layer supported by a porous substructure, confirming the formation of asymmetric membranes through the NIPS process. However, based on the present images, it is more accurate to describe the sublayer as a porous internal structure rather than as a clearly developed finger-like macrovoid morphology. In the neat PSM membrane (Figure 3A), the cross-section appears relatively dense with limited visible internal void development. The membrane containing 1 wt% TNT@OH (Figure 3B) shows a more open sublayer than neat PSM, while the 3 wt% membrane (Figure 3C) appears comparatively compact in the cross-sectional view despite its more porous surface. The 5 wt% membrane (Figure 3D) exhibits the most pronounced porous substructure among the presented samples, indicating stronger nonsolvent–solvent exchange and more distinct internal pore development. In contrast, the 7 wt% membrane (Figure 3E) exhibits a denser, more compact cross-section, which may be attributed to reduced phase-separation uniformity at higher nanoparticle loading, possibly due to partial aggregation of TNT@OH within the casting solution [20]. These FESEM observations suggest that TNT@OH incorporation modifies both surface and internal membrane morphology, although the effect is not strictly linear with filler loading. Moderate TNT@OH contents, especially around 3–5 wt%, appear to favor the formation of a more porous surface and a more developed internal structure, which can contribute to enhanced water permeability and improved antifouling behavior. At higher loading (7 wt%), the denser cross-sectional structure may reduce the effective transport pathways despite the presence of surface pores. Therefore, the membrane performance is likely governed by the combined influence of surface pore formation, sublayer compactness, and the hydrophilic effect of TNT@OH, rather than by any single morphological feature. Similar trends have been reported for TiO_2_- and nanofiller-modified polysulfone membranes, where moderate additive loading improves membrane morphology and performance, while excessive loading may hinder structural uniformity [12,18].

**Figure 4:**
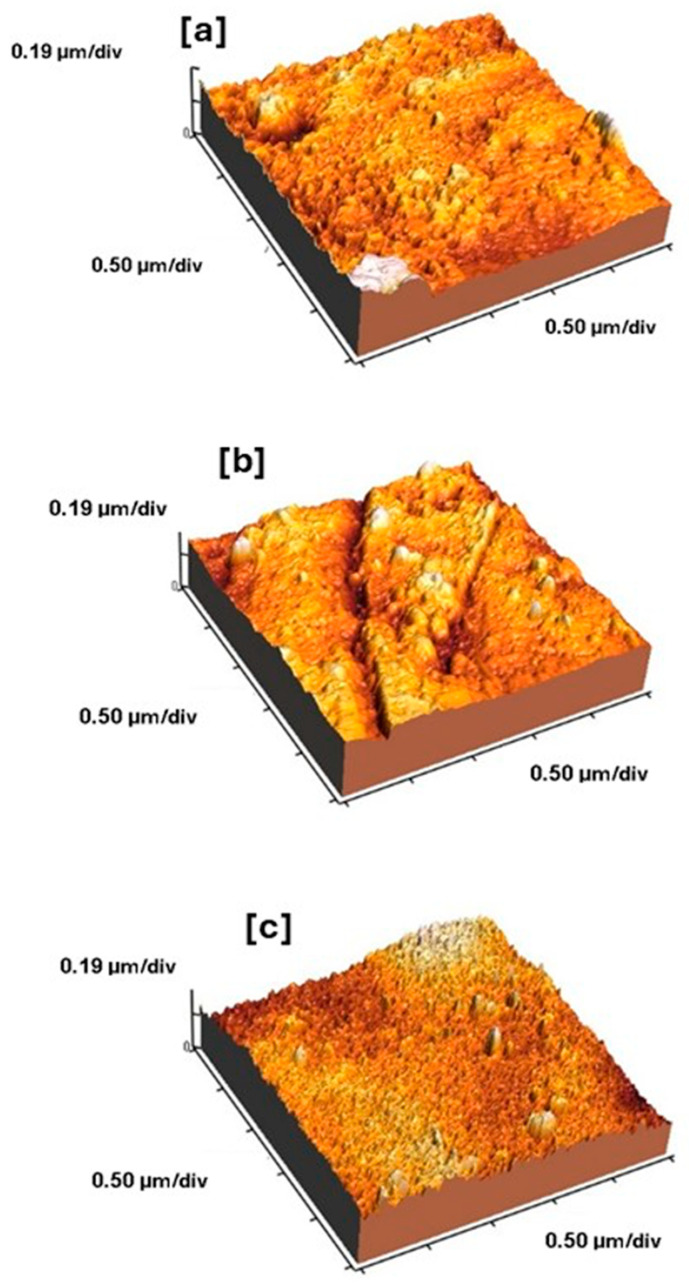
AFM topographical 3D images of (**a**) neat PSM, (**b**) PSM-TNT@OH 1%, and (**c**) PSMTNT@OH 5% membranes.

AFM 3D topographical images in Figure 4 provide quantitative and qualitative information about the membrane surface roughness. Neat PSM (a) displays a smooth surface with minimal height variation, which corresponds to lower roughness values. When 1% TNT@OH is incorporated (b), the surface becomes slightly rougher due to the presence of dispersed nanotubes, leading to an increase in effective surface area. At a 5% TNT@OH loading (c), the surface exhibits a more pronounced texture, with greater height differences, indicating increased roughness due to protruding nanotubes and surface heterogeneity. Surface roughness plays a dual role in membrane performance. A moderately rough surface increases the surface area, thereby enhancing water permeability. It also promotes better interaction with water molecules due to the presence of surface hydroxyl groups on TNT@OH, improving hydrophilicity. However, excessive roughness can lead to localized foulant deposition and irreversible fouling because peaks and valleys trap contaminants more easily. The 5% TNT@OH membrane demonstrates an optimal balance, providing enhanced water flux and antifouling behavior without excessive roughness that might exacerbate fouling [26]. This result aligns with previous studies showing that appropriate nanofiller loadings enhance both flux and flux recovery ratio due to the combined effects of increased hydrophilicity and optimal surface roughness. Overall, the FESEM and AFM results collectively confirm that TNT@OH incorporation significantly alters the membrane structure and surface properties, directly influencing permeability, hydrophilicity, and antifouling behavior. The 3% and 5% loadings are identified as optimal for achieving improved pore structure and surface characteristics without causing excessive nanoparticle aggregation or pore blockage.

**Figure 5:**
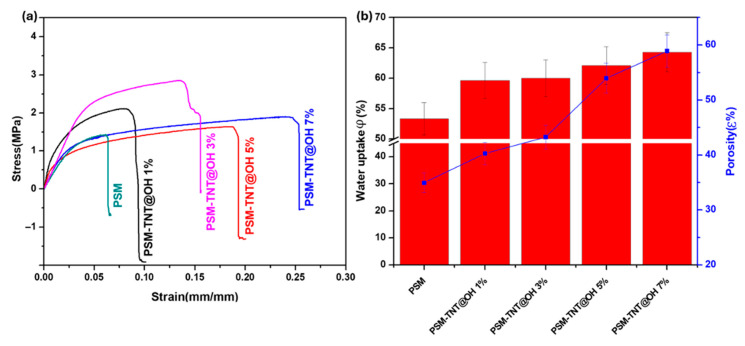
(**a**) Tensile stress–strain curves of neat PSM and composite membranes PSM-TNT@OH containing 1%, 3%, 5%, and 7% TNT@OH; (**b**) Water uptake and porosity (%) of neat PSM and the corresponding composite membranes.

The stress–strain curves in Figure 5a demonstrate that TNT@OH incorporation significantly influences the mechanical properties of the membranes. Neat PSM exhibits moderate tensile strength and low elongation at break, characteristic of brittle polymer membranes. The inclusion of TNT@OH at 1% and 3% increases tensile strength and ductility, indicating effective load transfer between the polymer matrix and the dispersed nanotubes. The best mechanical performance is observed at 5% loading, where both tensile strength and elongation are maximized, suggesting optimal dispersion and strong interfacial interactions between TNT@OH and the polymer chains through hydrogen bonding. At 7% loading, a slight decline in strength and ductility occurs, likely due to nanotube agglomeration, which can act as stress concentrators and defects. This trend aligns with studies on TiO_2_-modified polysulfone membranes, in which optimal nanofiller levels enhance mechanical stability, whereas excessive filler reduces performance due to aggregation [34,35].

Figure 5b shows a gradual increase in bulk membrane porosity (as determined by the gravimetric method) along with water uptake as the TNT@OH content increases. Even a 1% addition leads to noticeable improvement due to the hydrophilic –OH groups on the nanotubes. As the filler loading rises to 3% and 5%, water uptake and porosity increase significantly, indicating that TNT@OH accelerates solvent–non–solvent exchange during phase inversion, resulting in larger and more interconnected pores. At 7% loading, water uptake and porosity reach their highest values, but this may not correspond to the best filtration performance due to potential structural weaknesses and reduced selectivity caused by excessive porosity. Enhanced water uptake and porosity contribute to higher pure-water flux and improved antifouling properties, as water preferentially occupies hydrophilic sites on the membrane surface, thereby reducing foulant adhesion. These results align with recent studies showing that incorporating hydrophilic nanomaterials, such as TiO_2_ nanotubes, increases membrane permeability and antifouling performance by improving pore structure and surface wettability. Collectively, Figure 5 demonstrates that the addition of TNT@OH enhances both the mechanical and transport properties of the membranes up to an optimal loading of around 5%. At this level, membranes exhibit improved strength, flexibility, porosity, and hydrophilicity, all of which are advantageous for practical ultrafiltration applications. Excessive TNT@OH (7%) leads to reduced mechanical stability due to agglomeration and potential compromise of membrane selectivity despite higher porosity. These findings highlight the importance of optimizing filler loading to balance mechanical strength, permeability, and antifouling performance for advanced membrane design.

### 3.3. Hydrophilicity and Permeability

**Figure 6:**
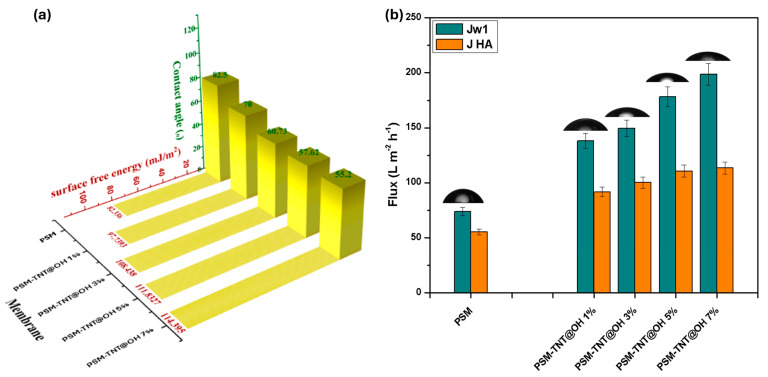
(**a**) Water contact angle and surface free energy of neat PSM and composite membranes PSM-TNT@OH with 1%, 3%, 5%, and 7% TNT@OH; (**b**) Pure water and humic acid (HA, 100 ppm) solution flux of neat PSM and the corresponding composite membranes.

Figure 6a illustrates the variation in water contact angle and surface free energy for neat PSM and TNT@OH-modified membranes. The neat PSM membrane exhibits the highest water contact angle (~82.3°) and lowest surface free energy, confirming its hydrophobic nature. As TNT@OH content increases, the contact angle decreases markedly, reaching approximately 72° at 1% loading, 60.73° at 3%, 57.62° at 5%, and 55.2° at 7%, indicating enhanced surface hydrophilicity due to hydroxyl groups on the nanotubes. Correspondingly, the surface free energy increases, which facilitates better interaction between water molecules and the membrane surface. This trend is consistent with the chemical structure of TNT@OH, in which abundant –OH groups enhance surface wettability by forming hydrogen bonds with water. The reduction in contact angles and increase in surface energy are most pronounced at 3% and 5% loadings, suggesting optimal nanotube dispersion within the polymer matrix. At 7% loading, a slight deviation from this trend may occur due to filler aggregation, which can reduce the effective number of accessible hydroxyl groups. Enhanced hydrophilicity is crucial for antifouling properties because water molecules preferentially hydrate the surface, forming a protective layer that reduces foulant adhesion.

Figure 6b shows the pure water flux (J_w1_) and humic acid (HA) flux for bare and composite membranes. Neat PSM exhibits the smallest flux for both water and HA solutions due to its dense structure and hydrophobic surface. With TNT@OH incorporation, both water and HA fluxes increase substantially, particularly for the 3% and 5% membranes. The improved flux can be related to the combined effects of rose porosity, surface hydrophilicity, and enhanced pore connectivity. At 7% loading, the pure water flux remains high; however, the HA flux shows a relatively smaller improvement, possibly due to partial pore blockage from nanoparticle agglomeration at high filler content. The flux improvement in the HA solution further demonstrates that TNT@OH-modified membranes exhibit better antifouling performance, as the hydrophilic surface hinders humic acid adsorption and facilitates easier water transport. Figure 6 clearly demonstrates that TNT@OH has a pronounced impact on both surface properties and filtration behavior of the membranes. Increasing the nanotube content progressively lowers the contact angle while raising the surface free energy, indicating enhanced surface hydrophilicity. These changes are directly associated with the observed increases in fluxes of pure water and humic acid solutions, indicating improved permeability and resistance to fouling. The most significant benefits occur at 3–5% TNT@OH, where the nanofiller is uniformly dispersed and effectively modifies the surface without the drawbacks of agglomeration. These findings reinforce that embedding hydroxylated titanium nanotubes into polysulfone membranes is a practical approach to producing advanced antifouling membranes with superior wettability, permeability, and resistance to organic fouling.

### 3.4. Rejection Performance and HA Adsorption

**Figure 7:**
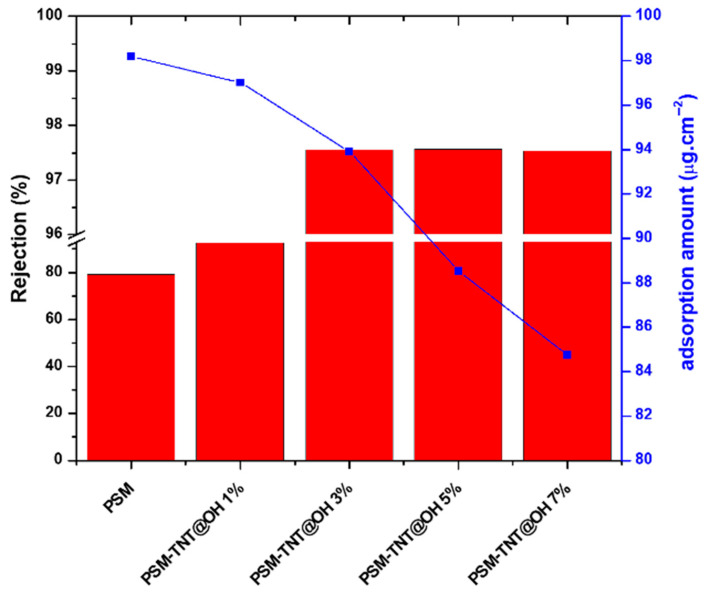
Rejection performance and adsorbed HA amount during ultrafiltration (UF) of humic acid (HA) solution using neat PSM and composite membranes PSM-TNT@OH containing 1%, 3%, 5%, and 7% TNT@OH.

Figure 6 and Figure 7 present the rejection efficiency and adsorbed humic acid (HA) amount of neat PSM and TNT@OH composite membranes during ultrafiltration. The neat PSM membrane shows the lowest HA rejection (~78%) and the highest adsorbed HA amount (~99 µg cm^−2^), reflecting its hydrophobic surface and limited ability to repel organic foulants. As TNT@OH content increases, rejection performance improves significantly, reaching ~97–98% for membranes containing 3–7% TNT@OH. This enhancement is related to the boosted hydrophilicity and surface free energy of TNT@OH, which improves water transport while minimizing foulant adhesion. The hydroxyl groups on TNT@OH create a hydration layer that acts as a physical barrier to HA adsorption, thereby reducing organic fouling. The adsorbed HA amount decreases progressively with higher TNT@OH loadings, dropping to ~85 µg cm^−2^ at 7% loading. These results demonstrate that TNT@OH incorporation not only enhances rejection but also effectively mitigates foulant deposition by altering surface chemistry and charge characteristics. The improved rejection and reduced adsorption at optimal loadings (3–5%) are consistent with previous studies, which show that the inclusion of TiO_2_-based nanomaterials in polysulfone membranes enhances hydrophilicity, porosity, and charge properties, leading to superior organic foulant resistance [22]. Additionally, similar findings have been reported for hydroxylated TiO_2_ nanotube-modified membranes, in which a hydration layer and reduced foulant–surface interactions synergistically improve antifouling behavior [18]. These observations confirm that the hydrophilic modification of polysulfone membranes with TNT@OH is an efficient strategy to enhance both rejection performance and long-term antifouling stability in ultrafiltration applications.

### 3.5. Antifouling Performance During Multi-Cycle Filtration

**Figure 8:**
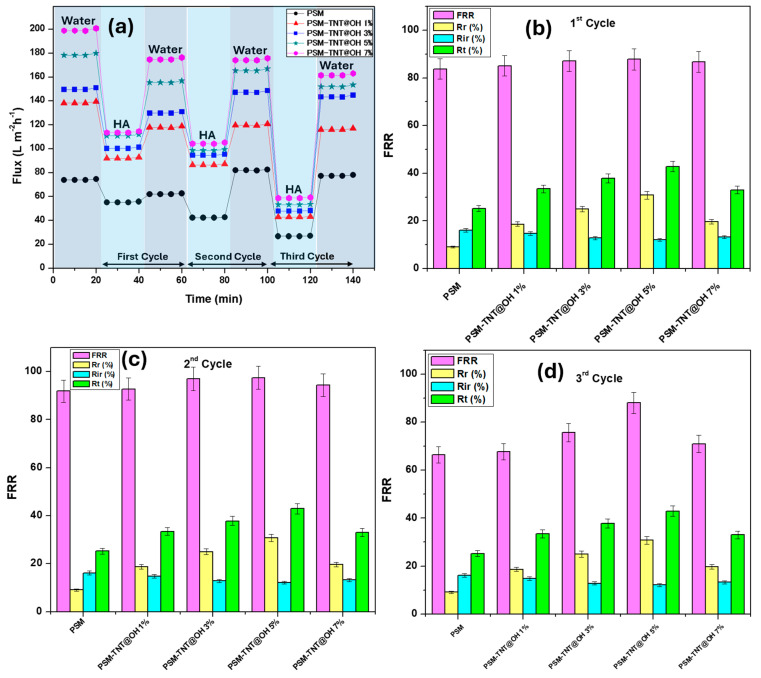
(**a**) HA flux of the prepared membranes over a 3-cycle filtration–cleaning process, along with corresponding values for flux recovery ratio (FRR), irreversible fouling ratio (Rir), reversible fouling ratio (Rr), and total fouling ratio (Rt); (**b**) 1st cycle, (**c**) 2nd cycle, and (**d**) 3rd cycle.

Figure 8 evaluates the antifouling properties of the membranes over three filtration–cleaning cycles using HA solution as the model foulant. Panel (a) shows that neat PSM suffers from significant flux decline in each cycle, while TNT@OH-modified membranes, particularly at 3% and 5% loadings, maintain higher flux and demonstrate better recovery after cleaning. Panels (b–d) quantify flux recovery ratio (FRR), reversible fouling ratio (R_r_), irreversible fouling ratio (R_ir_), and total fouling ratio (R_t_). Neat PSM exhibits the lowest FRR and highest R_ir_, confirming severe irreversible fouling due to its hydrophobic nature. With increasing TNT@OH content, FRR improves markedly, reaching above 85% for 3% and 5% membranes, while R_ir_ decreases substantially. These improvements are attributed to the enhanced hydrophilicity and smoother water channels created by TNT@OH, which reduce foulant adhesion and facilitate cleaning. At 7% loading, a slight decline in performance is observed, possibly due to filler agglomeration, which reduces effective surface modification. These trends align with reports that TiO_2_-based nanofillers reduce irreversible fouling and enhance cleaning efficiency by forming a hydration layer that hinders foulant deposition.

### 3.6. NOM Rejection and Water Flux Recovery

**Figure 9:**
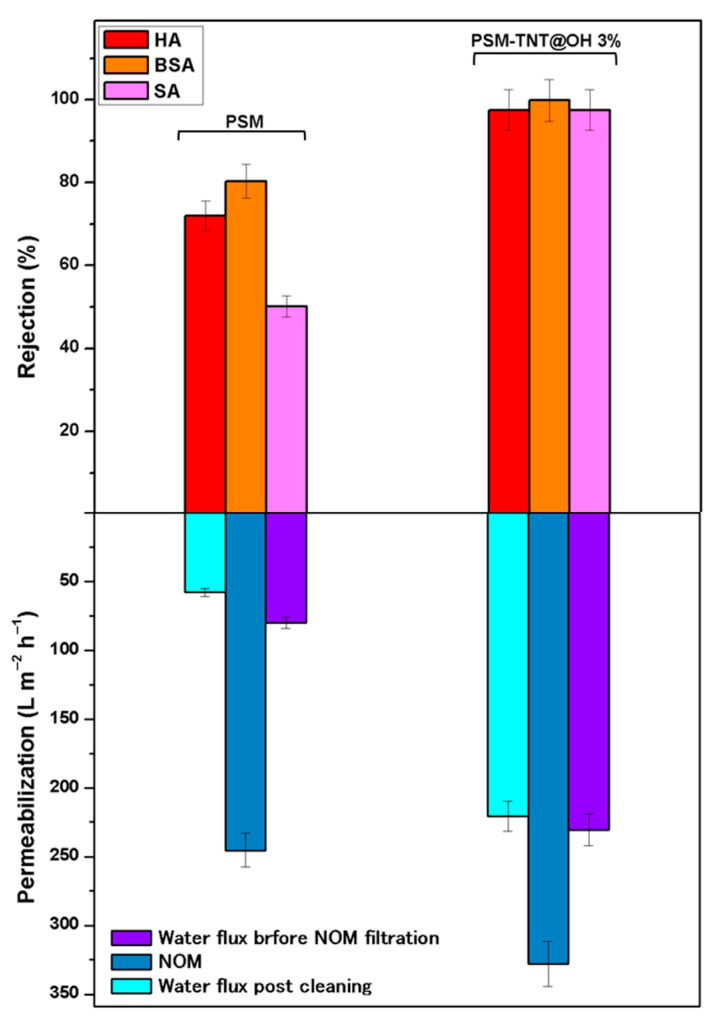
Rejection performance, natural organic matter (NOM) removal, and pure water flux measurements before and after ultrafiltration (UF) of NOM using neat PSM and composite membranes PSM-TNT@OH with 1%, 3%, 5%, and 7% TNT@OH.

Figure 9 compares the rejection of different NOM components, humic acid (HA), bovine serum albumin (BSA), and sodium alginate (SA), and water flux before and after ultrafiltration. The TNT@OH-modified membrane (3% loading) achieves significantly higher rejection for all foulants (~85–90%) compared to neat PSM (~60–70%), demonstrating its superior ability to block diverse NOM species. Pure water flux before filtration is higher for TNT@OH-modified membranes, while post-cleaning flux recovery is also markedly improved, indicating reduced irreversible fouling. The results confirm that TNT@OH enhances both permeability and foulant resistance due to its hydrophilic surface and uniform pore structure. These findings are consistent with recent studies demonstrating that TiO_2_ nanotube-modified membranes achieve improved NOM rejection and flux recovery due to their surface hydroxylation and improved dispersion in the polymer matrix. Together, Figure 8 and Figure 9 highlight the excellent antifouling performance and stability of TNT@OH composite membranes under repeated filtration cycles and when exposed to different organic foulants.

## 4. Comparative Studies

Table 1 presents a comparative assessment of the developed PSM–TNT@OH membranes against recently reported nanocomposite ultrafiltration membranes, highlighting key parameters such as hydrophilicity, permeability, rejection efficiency, and antifouling performance [20,36,37,38,39]. It is evident that conventional PSf/TiO_2_ nanoparticle-based membranes exhibit moderate improvements in water flux and rejection; however, their performance is often limited by nanoparticle agglomeration and insufficient long-term antifouling stability. For instance, GO/TiO_2_-modified membranes demonstrate high water flux (496.2 L m^−2^ h^−1^) and rejection (96.6%), but their flux recovery ratio (75.8%) remains comparatively lower, indicating partial irreversible fouling. Similarly, TiO_2_ nanotube-incorporated PES membranes show enhanced antifouling performance (FRR ≈ 93%), yet their organic matter rejection (53.9%) is relatively low, suggesting limited selectivity. Modified TNT/PSf systems further improve fouling resistance (FRR ≈ 93.5%) but exhibit reduced permeability and moderate salt-rejection performance. Other advanced systems, such as HNT-HFO/PES membranes, achieve excellent flux and near-complete flux recovery (>98%); however, they often require more complex fabrication processes and may face challenges in scalability. Likewise, MWCNT-based membranes exhibit significant permeability enhancement (~187% increase) and good antifouling properties, but their performance is sensitive to dispersion stability and potential nanoparticle leaching. In contrast, the PSM–TNT@OH membranes developed in this study demonstrate a more balanced and superior overall performance. The significantly reduced contact angle (~50°) confirms enhanced surface hydrophilicity, which directly contributes to improved antifouling behavior. Furthermore, the membranes achieve high humic acid rejection (~98%) along with a high flux recovery ratio (>85%), indicating reduced irreversible fouling and stable filtration performance. This improved performance can be attributed to the high density of hydroxyl groups and the unique tubular structure of TNT@OH, which promotes uniform dispersion, strong interfacial interactions, and the formation of a stable hydration layer. Overall, the comparison clearly demonstrates that the proposed PSM–TNT@OH membranes outperform many previously reported systems by offering an optimal combination of hydrophilicity, rejection efficiency, and antifouling stability, making them promising candidates for advanced water treatment applications.

## 5. Proposed Mechanism of Antifouling Enhancement

The improved antifouling performance of PSM-TNT@OH composite membranes can be attributed to several synergistic effects introduced by the hydroxylated titanium nanotubes. The surface –OH groups on TNT@OH increase membrane hydrophilicity, as evidenced by the reduced water contact angle and increased surface free energy. These hydroxyl groups form a hydration layer on the membrane surface, acting as a physical and energetic barrier against the adhesion of organic foulants. The enhanced hydrophilicity also promotes faster solvent–non-solvent exchange during phase inversion, resulting in membranes with higher porosity and more interconnected, finger-like pores, thereby increasing water permeability. The well-dispersed TNT@OH also enhances polymer–nanoparticle interactions through hydrogen bonding, thereby strengthening the membrane structure and improving its mechanical properties. Optimal TNT@OH loading (3–5%) yields the best balance among hydrophilicity, porosity, and mechanical stability. At these loadings, TNT@OH is uniformly dispersed, avoiding aggregation that may occur at higher concentrations. The crystalline anatase structure of TNT@OH, preserved after incorporation, further contributes to surface hydrophilicity and structural stability. During filtration, the hydration layer formed by surface –OH groups minimizes irreversible fouling by preventing foulant adhesion. Consequently, the flux recovery ratio is higher, and the irreversible fouling ratio is lower for TNT@OH-modified membranes than for neat PSM. These effects collectively lead to improved rejection of natural organic matter (NOM) and enhanced cleaning efficiency after repeated filtration cycles. Similar antifouling mechanisms have been reported for membranes modified with TiO_2_ nanotubes and other hydrophilic nanomaterials, confirming that such surface modifications enhance permeability, reduce fouling, and improve long-term operational stability.

## 6. Conclusions

This work demonstrates that hydroxylated titanium nanotube (TNT@OH) incorporation into polysulfone membranes significantly boosts their physicochemical and functional features. The addition of TNT@OH improved hydrophilicity, surface free energy, porosity, and mechanical strength, leading to superior water permeability and rejection of humic acid and other NOM components. Optimal TNT@OH loading (3–5 wt%) provided the best balance between structural stability and performance, achieving high flux recovery and low irreversible fouling over repeated filtration cycles. The improved antifouling behavior is primarily due to the hydration layer formed by surface hydroxyl groups, which minimizes foulant adhesion. These findings highlight TNT@OH-modified polysulfone membranes as promising candidates for advanced ultrafiltration applications, offering enhanced durability, antifouling resistance, and water purification efficiency. Future studies can explore scaling up the fabrication process and testing these membranes in real water treatment scenarios to confirm their practical applicability.

## Figures and Tables

**Table 1:** Comparative performance of TNT@OH-modified membranes with recently reported nanocomposite ultrafiltration membranes.

Membrane System	Verified Performance Values	Reference
GO/TiO_2_–PVP/PSf	Pure water flux: 496.2 L m^−2^ h^−1^; rejection: 96.6%; FRR: 75.8%	[36]
TiO_2_ nanotube/PES	Water contact angle: 69.5° at 1 wt% TiO_2_NT; PWF: 72 L m^−2^ h^−1^ bar^−1^; organic matter rejection: 53.9%; FRR: 93%	[37]
Modified TNT/PSf	Flux: 12.17 LMH; FRR: 93.5%; total fouling: 15.9%; MgCl_2_ rejection: 76.6%	[20]
HNT-HFO/PES	Contact angle: 50.30°; pure water flux: 516.33–640.82 L m^−2^ h^−1^; FRR: 100% by water washing; total flux recovery: >98%	[38]
TPGS/MWCNT-COOH/PES	PWP increase: ~187%; FRR: 89.3 ± 2.1% after protein fouling; 81.4 ± 3.5% after synthetic municipal wastewater fouling	[39]
PSM–TNT@OH	Contact angle: ~50°; HA rejection: ~98%; FRR: >85%	This study

## Data Availability

Data will be made available on request.
